# Global knowledge, attitude, and practice towards COVID-19 among pregnant women: a systematic review and meta-analysis

**DOI:** 10.1186/s12884-023-05560-2

**Published:** 2023-04-22

**Authors:** Abdolreza Sotoodeh Jahromi, Mohammad Jokar, Nader Sharifi, Benyamin Omidmokhtarloo, Vahid Rahmanian

**Affiliations:** 1 Zoonoses Research Center, Jahrom University of Medical Sciences, Jahrom, Iran; 2 Faculty of Veterinary Medicine, Karaj Branch, Islamic Azad University, Karaj, Iran; 3 Department of Public Health, Khomein University of Medical Sciences, Khomein, Iran; 4 Research Center for Social Determinants of Health, Jahrom University of Medical Sciences, Jahrom, Iran; 5 Department of Public Health, Torbat Jam Faculty of Medical Sciences, Torbat Jam, Iran

**Keywords:** COVID-19, Knowledge, Attitudes, Practice, Pregnant

## Abstract

**Background:**

Pregnant women form a specially vulnerable group due to unique changes in pregnancy, leading to a higher risk of getting a severe infection. As severe COVID-19 increases the risk of preeclampsia, preterm delivery, gestational diabetes, and low birth weight in pregnancy, there is a need to enhance pregnant women’s knowledge, attitudes, and practices to prevent these complications. This systematic review and meta-analysis aimed to determine their levels of knowledge, attitudes, and practice (KAP) regarding COVID-19 at the global level.

**Methods:**

The systematic literature search was conducted in the English language, including Google Scholar, Scopus, PubMed/MEDLINE, Science Direct, Web of Science, EMBASE, Springer, and ProQuest, from the occurrence of the pandemic until September 2022. We used The Newcastle Ottawa scale for cross-sectional studies checklist to evaluate the risk of bias in the studies. Data were extracted by a Microsoft Excel spreadsheet and analyzed by STATA software version 14. We also employed Cochran Q statistics to assess the heterogeneity of studies and utilized Inverse variance random-effects models to estimate the pooled level of pregnant women’s KAP towards COVID-19 infection prevention.

**Results:**

Based on the preferred reporting items for systematic reviews and meta-analyses (PRISMA) and inclusion criteria, 53 qualified studies were acquired from several countries. In total, 51 articles (17,319 participants) for knowledge, 15 articles (6,509 participants) for attitudes, and 24 articles (11,032 participants) for practice were included in this meta-analysis. The pooled good knowledge, positive attitude, and appropriate practice in pregnant women were estimated at 59%(95%CI: 52–66%), 57%(95%CI: 42–72%), and 53%(95%CI: 41–65%), respectively. According to subgroup analysis, the level of knowledge, attitude, and practice were 61%(95%CI: 49–72), 52%(95%CI: 30–74), and 50%(95%CI: 39–60), respectively, in Africa, and 58.8%(95%CI: 49.2–68.4), 60%(95%CI: 41–80) and 60% (95%CI: 41–78), respectively, in Asia.

**Conclusion:**

The Knowledge, attitude, and practice towards COVID-19 infection prevention in pregnant women were low. It is suggested that health education programs and empowerment of communities, especially pregnant women, about COVID-19 continue with better planning. For future studies, we propose to investigate the KAP of COVID-19 in pregnant women in countries of other continents and geographical regions.

**Supplementary Information:**

The online version contains supplementary material available at 10.1186/s12884-023-05560-2.

## Background

The WHO declared the pandemic caused by COVID-19 as a public health emergency of international concern in January 2020 [1]. As of 02 October 2022, it has resulted in 623,268,353 confirmed cases of COVID-19 and 6,549,980 deaths globally [2]. Over time, new aspects of the effect of this virus on different body organs were identified and reported. Studies showed its impact on the digestive system, nervous system, skin, smell, cardiovascular system, liver, kidney, and eyes [3–6]. In addition to physical symptoms, the psychological burden of COVID-19 patients was heavy and persistent. So, the ongoing psychological trauma of the survivors of COVID-19 was highlighted in health care [7]. As of March 2021, there were 80 reported maternal deaths due to COVID-19 in the United States, and as of October 6, 2021, 1,637 COVID-19 infections and 15 deaths were reported in Mississippi [8].

On the other hand, pregnant women are more vulnerable, especially in the case of emerging infections, due to physiological and immunological changes [9, 10]. They are at risk of contracting the disease because of the weakness of the immune system and being in general society [11]. Changes caused by disasters and crises harm women's health [12]. Moreover, the level of anxiety and stress during the COVID-19 pandemic is high, so women are worried about their babies getting infected and seeking prenatal care [13, 14]. The most common complications in pregnancy include acute respiratory distress, disseminated intravascular coagulation, renal failure, bacterial infection, sepsis, need for mechanical ventilation, fetal death, and preterm delivery [15, 16]. The type of delivery in affected pregnant women depends on the conditions of the fetus, mother, and cervix. Thus, infection with COVID-19 alone does not determine the type of delivery [17]. Furthermore, COVID-19 can also affect children and cause systemic disease with several internal organ involvements [18].

In a systematic review study, Turan et al*.* showed that increasing age, obesity, diabetes, D-dimer levels, and interleukin-6 were effective in predicting pregnancy outcomes at the time of COVID-19, leading to a rise in premature birth and cesarean section. Also, vertical transmission may be possible, although it has not been proven [19]. In another study, Simsek et al. reported that COVID-19 has a harmful effect on pregnancy [20]. The association of severe COVID-19 during pregnancy with preeclampsia, premature birth, gestational diabetes, and low birth weight was reported [21].

Considering the vulnerability of pregnant women, the availability of fully effective vaccines in preventing infection, and the lack of definitive treatment, it is suggested that prevention is possible by increasing the knowledge of society to apply the correct health principles and physical distance to prevent its prevalence. According to a study in Ethiopia, maternal age, educational levels, husband educational levels, underlying disease, and sociocultural and demographic features had an influence on the KAP of COVID-19 in pregnant women [22]. Although there are numerous studies about the KAP of pregnant women in the prevention of COVID-19, their findings are not consistent with each other in some cases. Therefore, an overall understanding of KAP on the prevention behaviors of COVID-19 in pregnant women is essential for health system policymakers and stakeholders to design prevention programs. As a result, this study aims to determine the level of knowledge, attitudes, and preventive actions of pregnant women regarding COVID-19 at the global level.

## Methods

This study was conducted according to Preferred Reporting Items for Systematic Reviews and Meta-Analyses guidelines [23, 24]. In addition, its executive protocol was registered in the international prospective register of systematic reviews (PROSPERO) with code [CRD42022351552],(https://www.crd.york.ac.uk/prospero/display_record.php?RecordID=351552).

### Search strategy

We searched all articles published in the English language, including Google Scholar, Scopus, PubMed/MEDLINE, Science Direct, Web of Science, EMBASE, Springer, and ProQuest, from the occurrence of the pandemic until September 2022.

**Fig. 1:**
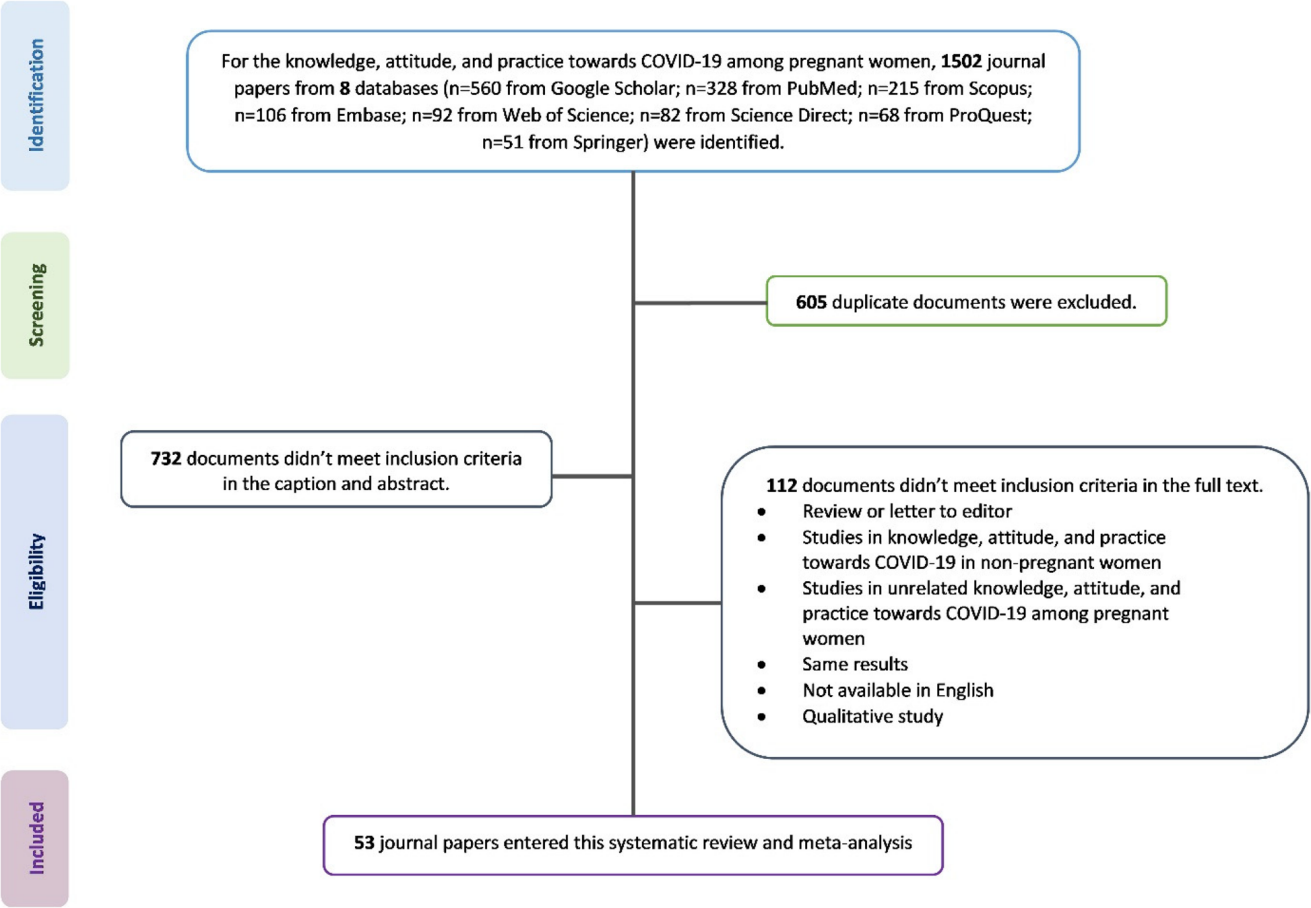
PRISMA flowchart presenting the selection of articles analyzed in this systematic review and meta-analysis

The search method was performed using MeSH terms in combination or separately using “AND” and “OR” functions (supplementary Table 1). The references of the found articles were also examined to increase the sensitivity. The processes of searching and selecting related articles are shown in the PRISMA flowchart (Fig. 1).

### Eligibility criteria

Databases were searched based on the mentioned strategy. Then, the collected articles were carefully reviewed in terms of the desired epidemiological parameters and the inclusion criteria:

1. All Cross-sectional studies that reported data on COVID-19 knowledge and attitudes and practices, as well as studies on KAP in COVID-19 in pregnant women.
2. All articles published in the English language from the occurrence of the pandemic until September 2022.
3. All articles whose full text was accessible.
4. Articles in which the subjects were selected based on random sampling or census.

Exclusion criteria.

1. Articles whose population was other than pregnant women (such as the general population, health care workers, and students).
2. Articles published in languages other than English.
3. Studies except for observational studies, such as reviews, case series, and short communication.

### Quality assessment (Risk of bias)

In this study, we used the modified Newcastle Ottawa scale for cross-sectional studies checklist to evaluate the risk of bias (internal validity) of the studies. The Newcastle–Ottawa Scale (NOS) is an ongoing collaboration between the Australian universities of Newcastle and Ottawa, Canada. This scale has been developed to evaluate the quality of non-randomized studies with its design, content, and ease of use to combine quality assessments in the interpretation of meta-analytic results. In this scale, studies are evaluated and graded based on three points of view, each of which includes subsections: a) Selection of study groups (including representativeness of the sample, sample size, ascertainment of exposure, and non-respondents), b) Ability to compare groups (the subjects in different outcome groups are comparable, based on the study design or analysis, and confounding factors), and c) determining the exposure or outcome of interest (assessment of the outcome and statistical test). The goal of the Newcastle–Ottawa Scale (NOS) is to develop a simple and convenient tool to assess the quality of non-randomized studies used in a systematic review.

The title of the journal and the names of the authors is apparent for the reviewers to measure the quality assessment of included studies. First, the full text of the article was read carefully by the first referee, and then the quality assessment checklist was completed and scored. The same steps were done independently by the second referee. Disagreements were discussed in a group discussion session. The range of scores is 0–10, calculated based on the checklist for each study. So, we determine the risk of bias for articles divided into three categories with low risk (8–10), medium risk (5–7), and high risk (0–5) [25].

### Data extraction

At first, all selected articles were entered into EndNote X8 software (Thomson Reuters, New York, USA), and duplicate articles were removed. Then, two team members (MJ and VR) reviewed the selected titles and abstracts and excluded irrelevant articles from the study.

We tried to select articles related to the research topic and compatible with descriptive and cross-sectional studies based on working methods. After choosing the appropriate ones according to the study objectives, the final selection was made through group discussion. Then, the articles were entered into the next processes for qualitative evaluation and information extraction.

The data from the articles include the name of the author(s), year of study, type of study, sample size, geographical region of the study, and a good level of knowledge, good attitude, and appropriate practice towards COVID-19, which were extracted.

In this study, the knowledge, attitude, and practice about Covid-19 were as follows:

#### Knowledge

Containing disease symptoms, route of transmission, incubation period and isolation period, and ways to prevent COVID-19 were used to assess knowledge. A good level of knowledge means an above-average score.

#### Attitude

It included the individual's agreement or desire to participate in the fight against the epidemic of COVID-19, as well as the trust in the government and her companions in winning the battle against the COVID-19 pandemic. A score above the average level is recognized as a good attitude in the control and management of COVID-19.

#### Practice

It was defined as preventing infection and implementing prevention recommendations, such as maintaining physical distance, hand hygiene, wearing a mask, avoiding crowded places or social events, and isolation and quarantine to prevent the spread of COVID-19. Those whose score is average or higher are considered to be appropriate practices.

### Statistical analysis

In this meta-analysis study, we performed statistical analyzes employing the STATA software (version 14.). We also used Inverse variance and Cochran Q statistics to evaluate the heterogeneity of studies. Low, medium, or high heterogeneity was considered as I^2^ test statistics. Values < 50%, 50%-80%, and > 80% were defined as low, moderate, and high heterogeneity, respectively [26]. Due to heterogeneity, the Dersimonian and Liard random-effects models were used in the current paper [27].

To evaluate the source of heterogeneity, univariate and multivariable meta-regression methods were used, as well as subgroup analysis [24]. In the analysis of the subgroups, the level of appropriate knowledge, positive attitude, and appropriate practice regarding preventive behaviors toward COVID-19 were estimated based on geographical areas.

We used the Funnel plots and Egger's regression test to check the existence of publication bias. On a condition of confirmation of publication bias, the trim-and-fill method was used to estimate the number of censored studies and correct the final estimate [28].

In addition, we used Arc GIS 10.3 software to visualize the geographic distribution of appropriate knowledge, positive attitudes, and appropriate practice according to continents and countries.

## Results

### Search results and eligibility studies

A total of 1,502 articles were reviewed by searching the seven mentioned databases based on the inclusion criteria. In the next step, 605 articles were excluded due to duplicates and 732 articles due to a lack of inclusion criteria in the abstract and title. Furthermore, 112 studies were excluded based on the exclusion criteria, such as the type of study, non-pregnant target group, and lack of access to the full text of the article. Finally, 53 studies, including 52 studies for knowledge [29–80], 15 studies for attitude [32, 36, 46–48, 50, 51, 55, 57, 66, 71, 73, 74, 77, 79], and 24 studies for practice [32, 40, 46, 48, 50, 52, 55, 57, 58, 62, 64, 65, 67–69, 71, 73–79, 81] were included in this systematic review and meta-analysis (Fig. 1).

### Characteristics of the eligible studies

Total eligible studies include 53 journal articles. In terms of evaluating the quality assessment of included studies, 44 studies with low risk of bias and nine studies with moderate risk of bias were scored based on the NOS quality scale, and no one was included in the high risk of bias category (Table 1). Based on the continent, 30 studies were conducted in Asia, 21 in Africa, and one in North America (Tables 1 and 2). In terms of the type of study, all studies were conducted in a cross-sectional design (Table 1).

**Table 1:** The article met the eligibility criteria of this systematic review and meta-analysis

Authors Name	Year	Country	Total number	Study type	Good knowledge %	Good Attitude %	Appropriate practice%	QA
Ahlers-Schmidt, C.R [29]	2020	USA	114	CS	35.9	NA	NA	9
Kiftia, M [30]	2022	Indonesia	138	CS	65.9	NA	NA	8
Lee, TY [31]	2020	China	161	CS	77.5	NA	NA	8
Sukontrakoon, S [32]	2022	Thailand	283	CS	75.62	8.48	88.34	8
Septiasari, RT [33]	2021	Indonesia	53	CS	24.5	NA	NA	4
Tindaon, RL [34]	2022	Indonesia	39	CS	97.4	NA	NA	4
Novelia, S [35]	2021	Indonesia	112	CS	58	NA	NA	5
Oktaviani, M [36]	2022	Indonesia	100	CS	55	57	NA	6
Rahmawati, VE [37]	2021	Indonesia	72	CS	87.5	NA	NA	5
Sajid, A [38]	2020	Pakistan	600	CS	85.69	NA	NA	9
Sultana, R [39]	2021	Pakistan	400	CS	33.3	NA	NA	8
Abdulla, TN [40]	2021	Iraq	400	CS	72	NA	32.75	9
Hakiki, M [41]	2022	Indonesia	35	CS	11	NA	NA	4
Tamtiana, NK [42]	2021	Indonesia	110	CS	49.1	NA	NA	5
Aghababae, S [43]	2020	Iran	225	CS	93.8	NA	NA	6
Indumathi, P [44]	2022	India	325	CS	50.5	NA	NA	7
El Taha, L [45]	2021	Lebanon	163	CS	81.5	NA	NA	5
Izhar, R [46]	2021	Pakistan	376	CS	39.4	62.8	30.9	8
Kundaryanti, R [47]	2021	Indonesia	73	CS	54.8	41.1	NA	5
Alsafi, R [48]	2022	Saudi Arabia	1574	CS	48.5	77.4	94.7	9
Jhirwal, M( 49)	2022	India	109	CS	94.4	NA	NA	7
Deep Kamal [50], SC	2022	India	506	CS	75.3	73.9	92.7	8
MM, K [51]	2021	India	505	CS	97.2	92.7	NA	8
Kaream, AK [52]	2021	Iraq	150	CS	28.7	NA	28	6
Rahayuningsih, FB [53]	2021	Indonesia	40	CS	15	NA	NA	4
Bahrum,SW [54]	2021	Indonesia	30	CS	46.7	NA	NA	4
Hamzehgardeshi, Z [55]	2021	Iran	318	CS	46.9	34.9	66.7	6
Maharlouei, N [56]	2020	Iran	540	CS	44.8	NA	NA	8
Ali, HA [57]	2022	Egypt	415	CS	75.4	95	43.6	8
Temesgan, WZ [58]	2022	Ethiopia	678	CS	62.2	NA	44.8	9
Aboma, D [59]	2021	Ethiopia	232	CS	63	NA	NA	8
Elhameed E, [60]	2022	Egypt	290	CS	15	NA	NA	7
Abdus-Salam, RA [61]	2021	Nigeria	380	CS	15	NA	NA	9
Burodo A [62]	2022	Nigeria	394	CS	98.7	NA	19.1	9
Metwally, HM [63]	2020	Egypt	370	CS	57.6	NA	NA	7
Omozuwa [64]	2021	Nigeria	420	CS	46.9	NA	77.1	8
Ayele, A [65]	2020	Ethiopia	405	CS	46.8	NA	47.6	8
Degu [66]	2021	Ethiopia	403	CS	52.1	52.6	NA	8
Kassie BA, [67]	2021	Ethiopia	422	CS	55	NA	47.4	9
Kumbeni, M [68]	2021	Ghana	527	CS	85.6	NA	46.6	9
Fikadu, Y [69]	2021	Ethiopia	403	CS	54.8	NA	76.2	8
Adegoke, J [70]	2020	Nigeria	382	CS	86.65	NA	NA	8
Aduloju, O [71]	2021	Nigeria	423	CS	87.2	74.5	79.2	8
Omoronyia, E [72]	2021	Nigeria	284	CS	43.3	NA	NA	7
West, B [73]	2021	Nigeria	253	CS	81.4	20.2	26.5	7
Hoque, A [74]	2021	South Africa	346	CS	43.5	30	76	8
Theuring, S [75]	2021	Uganda	648	CS	32.8	NA	21.4	8
Besho, M [76]	2021	Ethiopia	415	CS	75.4	NA	43.6	8
Silesh, M [22]	2021	Ethiopia	396	CS	70.5	87.6	56.1	8
Nwafor, J [78]	2020	Nigeria	284	CS	60.9	NA	30.3	7
Zeleke, A [79]	2022	Ethiopia	538	CS	67.3	46.7	51.1	9
Anikwe [80]	2021	Nigeria	460	CS	43.5	NA	NA	8
Belayneh, M [81]	2021	Ethiopia	458	CS		NA	53	8

*CS* Cross-Sectional, *QA* Quality assessment, *NA* Not applicable

**Table 2:** The results of subgroup analysis based on country and continent for knowledge, attitude, and practice for COVID-19 in pregnant women

type	grouping		No. studies	Sample size	Overall frequency (95%CI)	Heterogeneity		
						**χ**^**2**^	***P*-value**	**I**^**2**^** (%)**
**Knowledge**	Continent	Africa	21	9353	61(49–72)	4479.4	< 0.001	99.6%
		Asia	30	7852	58.8(49.2–68.4)	4036.7	< 0.001	99.3%
		North America	1	114	36(27–45)	NA	NA	NA
	Country	China	1	161	77.5(71–84)	NA	NA	NA
		Egypt	3	2520	49.3(12–86.6)	429.6	< 0.001	99.5%
		Ethiopia	9	4177	60.8(54.7–67)	131.0	< 0.001	93.9%
		Ghana	1	527	85.6(82.6–88.6)	NA	NA	NA
		India	4	1445	79.5(61.2–97.7)	351.1	< 0.001	99.1%
		Indonesia	11	802	51.6(33.3–69.8)	449.2	< 0.001	97.8%
		Iran	3	1083	61.9(26.7–97)	425.1	< 0.001	99.5%
		Iraq	2	550	50.4(8–92.2)	100.3	< 0.001	99.0%
		Lebanon	1	163	81.5(75.5–87.5)	NA	NA	NA
		Nigeria	9	5605	62.6(41.6–83.7)	2879.2	< 0.001	99.7%
		Pakistan	3	1376	52.8(16.1–89.6)	493.4	< 0.001	99.6%
		Saudi Arabia	1	1574	48.5(46–51)	NA	NA	NA
		South Africa	1	346	43.5(38–48.7)	NA	NA	NA
		Thailand	1	283	75.6(70.6–80.6)	NA	NA	NA
		Uganda	1	648	32.8(29.2–36.4)	NA	NA	NA
		USA	1	114	35.9(27.1–44.7)	NA	NA	NA
**Attitude**	Continent	Africa	6	2359	52(30–74)	765.4	< 0.001	99.3%
		Asia	9	4150	60(41–80)	2498.9	< 0.001	99.7%
	Country	Thailand	1	283	8.5(5.2–11.7)	NA	NA	NA
		Indonesia	2	173	49.3(33.7–64.9)	4.38	0.036	77.2%
		Pakistan	1	376	62.8(58.9–67.8)	NA	NA	NA
		Saudi Arabia	1	1574	77.4(75.3–79.5)	NA	NA	NA
		India	2	1011	83.4(64.9–98.01)	68.6	< 0.001	98.5%
		Iran	1	318	34.9(29.7–40.1)	NA	NA	NA
		Egypt	1	415	95(92.9–97.1)	NA	NA	NA
		Ethiopia	3	1337	62.3(34.7–89.9)	276.4	< 0.001	99.3%
		Nigeria	2	676	47.4(5.08–99.01)	271.4	< 0.001	99.6%
		South Africa	1	346	30(25.2–34.8)	NA	NA	NA
**Practice**	Continent	Africa	16	7010	50(39–60)	1382.2	< 0.001	98.9%
		Asia	8	4022	60(41–78)	1928.9	< 0.001	99.6%
	Country	Thailand	1	283	88.3(84.6–92.1)	NA	NA	NA
		Iraq	2	550	31.2(26.9–35.6)	1.2	0.275	16.0%
		Pakistan	1	376	30.9(26.2–35.6)	NA	NA	NA
		Iran	1	318	66.7(61.5–71.9)	NA	NA	NA
		Egypt	1	415	43.6(38.8–48.4)	NA	NA	NA
		Ethiopia	8	3715	52.5(44.8–60.1)	165.2	< 0.001	95.8%
		Nigeria	5	1774	46.5(19.3–73.6)	760.9	< 0.001	99.5%
		Ghana	1	527	46.6(42.3–50.9)	NA	NA	NA
		South Africa	1	346	76(71.5–80.5)	NA	NA	NA
		Uganda	1	648	21.4(18.2–24.6)	NA	NA	NA
		India	1	506	92.7(90.4–95)	NA	NA	NA
		Saudi Arabia	1	1574	94.7(93.6–95.8)	NA	NA	NA

*NA* Not applicable

### Pooled good knowledge about COVID-19

A total of 17,319 pregnant women were examined to estimate the level of good knowledge of COVID-19, which included 30 studies in Asia (7852 people), 21 studies in Africa (9353 people), and one study in America (114 people).

**Fig. 2:**
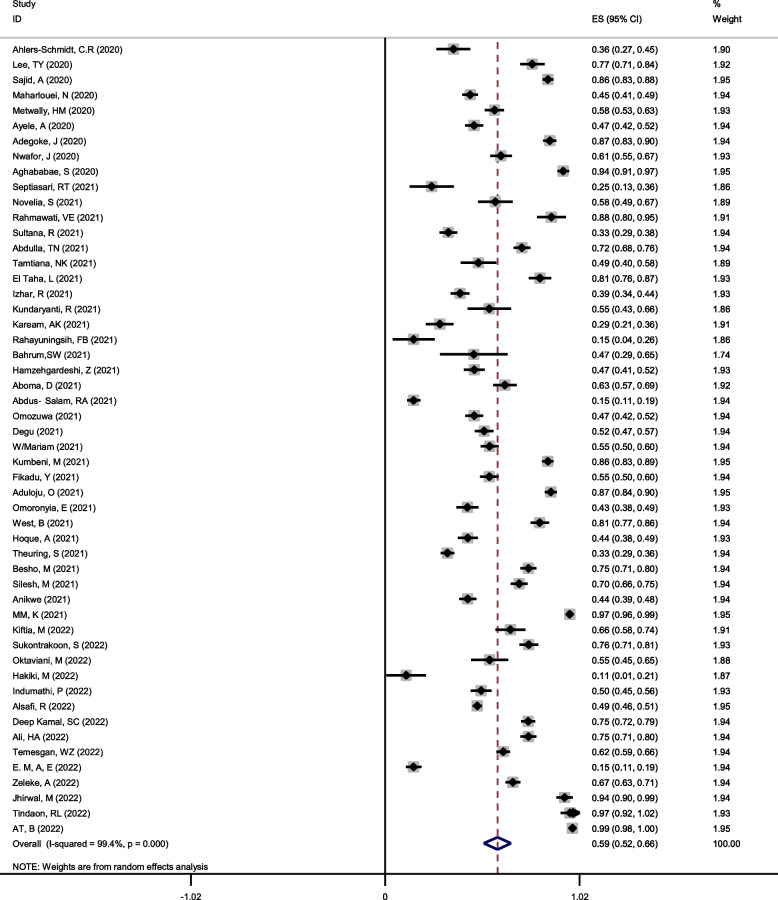
Forest plot of Mantel –Hanenszel random effect meta-analysis for good knowledge of COVID-19 among pregnant women

The overall good knowledge among pregnant women, using the random effect model with Mantel-Hanenszel heterogeneity, was estimated at 59% (95%CI: 52–66%) (Q statistic = 8632.71, d.f. = 51, *p* < 0.0001, I^2^ = 99.4%), as shown in Fig. 2.

Since the heterogeneity between studies was high, univariate and multivariable meta-regression methods were employed to investigate the cause and source of heterogeneity. In this regard, univariate meta-regression indicated that the country with a coefficient of 0.02281 might be its cause, which means that the percentage of good knowledge for COVID-19 can increase by 0.02281 with the change of the country, as demonstrated in Table 3.

**Table 3:** Univariate and multivariable meta-regression to find possible causes of heterogeneity between studies included in the meta-analysis

Type	Possible cause of heterogeneity	Univariate		Multivariable	
		**Coefficient (95%CI)**	***P*-value**	**Coefficient (95%CI)**	***P*-value**
**Knowledge**	Continent	-0.040(-0.162, 0.082)	0.510	-0.124(-0.283, 0.034)	0.123
	Country	0.022(0.016, 0.061)	0.041	0.010(-0.013, 0.0341)	0.123
	Risk of bias	-.075(-0.243, 0.092)	0.372	-0.086(-0.317, 0.143)	0.453
	Year	0.003(-0.096,0.103)	0.942	-0.007(-0.112, 0.097)	0.885
	Sample size	0.0005(-0.0002, 0.0003)	0.710	-0.0001(-0.0004, 0.0002)	0.497
**Attitude**	Continent	-0.085(-0.397, 0.226)	0.565	-0.410(-1.04, 0.225)	0.178
	Country	0.008(-0.046, 0.063)	0.748	0.052(-0.056, 0.160)	0.306
	Risk of bias	0.079(-0.056, 0.215)	0.229	0.082(-0.155, 0.319)	0.453
	Year	0.045(-0.270, 0.360)	0.762	-0.077(-0.493, 0.338)	0.685
	Sample size	0.0002(-0.0001, 0.0007)	0.167	0.00008(-0.0005, 0.0007)	0.776
**Practice**	Continent	-0.100(-0.309, 0.107)	0.325	-0.129(-0.592, 0.333)	0.563
	Country	-0.011(-0.049, 0.026)	0.541	0.007(-0.075,0.089)	0.859
	Risk of bias	-0.072(-0.437, 0.292)	0.683	-0.06113(-0.535, 0.413)	0.789
	Year	0.114(-0.051, 0.281)	0.167	0.05616(-0.173, 0.285)	0.614
	Sample size	0.0003(-0.00005, 0.0006)	0.091	0.0002(-0.0002, 0.0006)	0.296

**Fig. 3:**
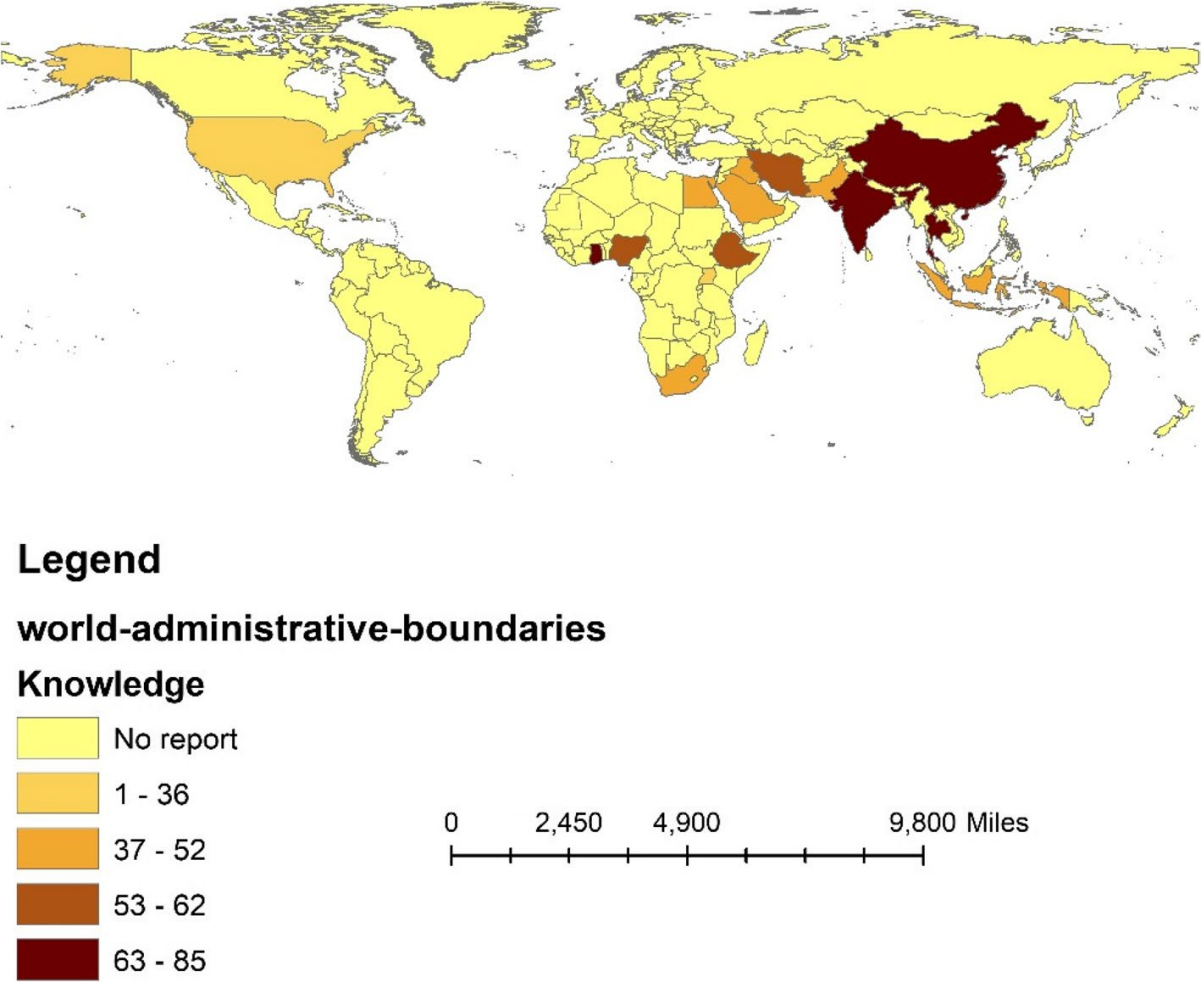
The percentage of good knowledge of COVID-19 among pregnant women based on countries

Based on the results of the subgroup analysis, the level of good knowledge about COVID-19 in pregnant women in Africa and Asia was estimated to be 61% (95%CI: 49–72) and 58.8% (95%CI: 49.2–68.4) (between-group *p*-value = 0.024), respectively. In regard to country, this level of knowledge was reported in Uganda at 32.8% (95%CI: 29.2–36.4) and Ghana at 85.6% (95%CI: 82.6–88.6) (between-group *p*-value < 0.001), as displayed in Table 3 and Fig. 3.

### Pooled good attitudes toward COVID-19

A total of 15 studies with 6509 people, including nine studies in Asia (4150 people) and six studies in Africa (2359 people), were included for attitude analysis.

**Fig. 4:**
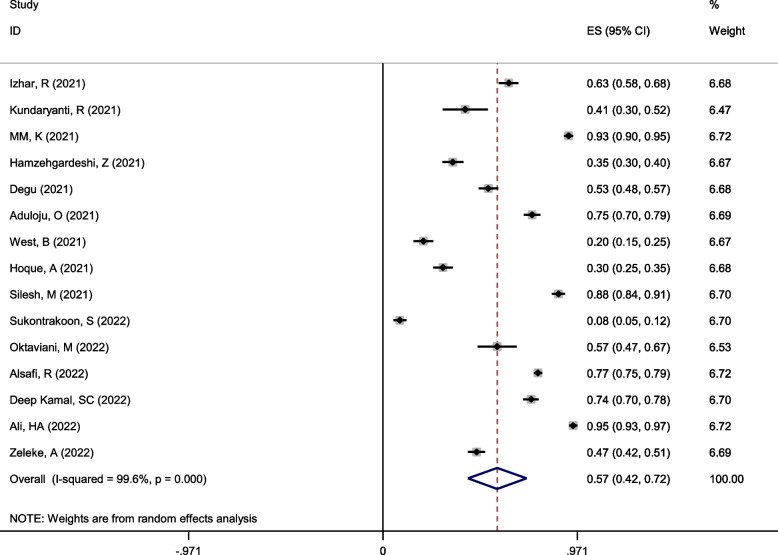
Forest plot of Mantel –Hanenszel random effect meta-analysis for good attitude towards COVID-19 among pregnant women

Using the random effect model with Mantel-Hanenszel heterogeneity, the overall good attitude among pregnant women was estimated at 57% (95%CI: 42–72%) (Q statistic = 3512.77, d.f. = 14, *p* < 0.0001, I^2^ = 99.6% %), as depicted in Fig. 4.

The results of univariate and multivariable meta-regression analysis showed that none of the variables of the continent, country, quality of studies, year of study, and sample size were possible causes of heterogeneity (*p* > 0.05), as shown in Table 3.

**Fig. 5:**
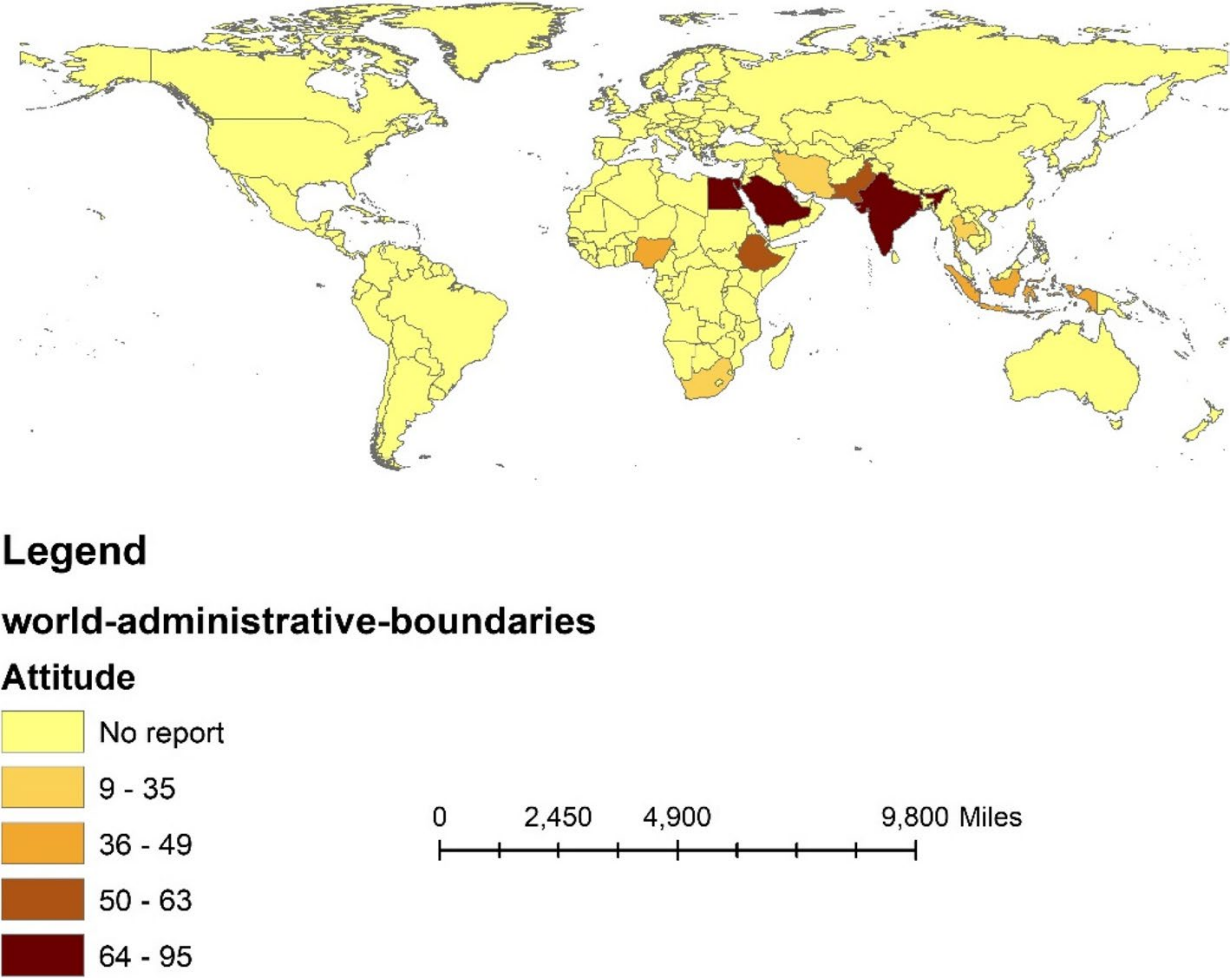
The percentage of positive attitudes towards COVID-19 among pregnant women based on the continent

Based on subgroup analysis, the level of positive attitude in Asia and Africa was estimated to be 60% (95%CI: 41–80) and 52%(95%CI: 30–74) (between-group *p*-value < 0.001), respectively, as demonstrated in Table 2 and Fig. 5.

### Pooled appropriate practice toward COVID-19

A total of 24 studies contained 11,032 pregnant women, including 16 studies in Africa (7010 people) and eight studies in Asia (4022 people).

**Fig. 6:**
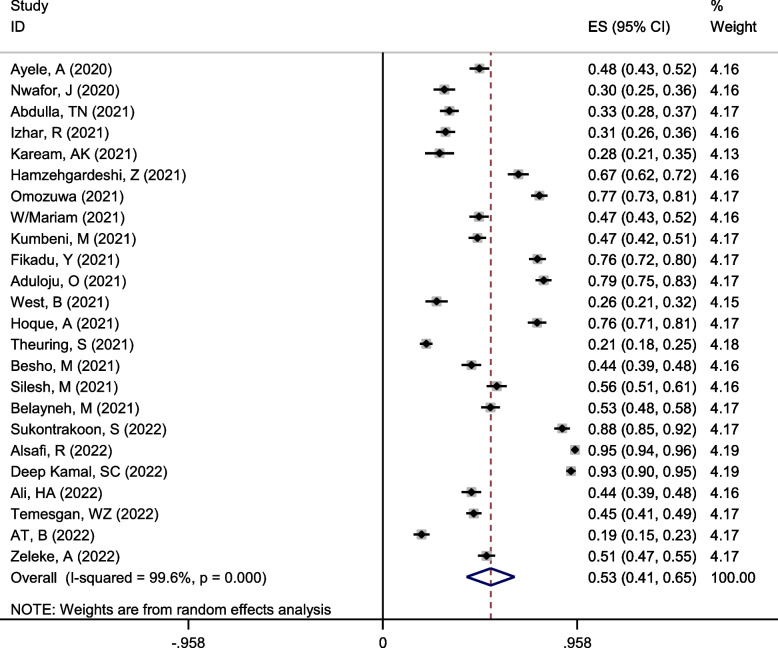
Forest plot of Mantel –Hanenszel random effect meta-analysis for appropriate practice towards COVID-19 among pregnant women

Employing a random effect model with Mantel-Hanenszel heterogeneity, the pooled appropriate practice was estimated at 53% (95%CI: 41–65%) (Q statistic = 5968.39, d.f. = 23, *p* < 0.0001, I^2^ = 99.6% %), as presented in Fig. 6.

Univariate and multivariable meta-regression analysis was performed to find the source of heterogeneity. Table 3 shows that none of the variables of the continent, country, quality of studies, year of study, and sample size are possible causes of heterogeneity (*p* > 0.05).

**Fig. 7:**
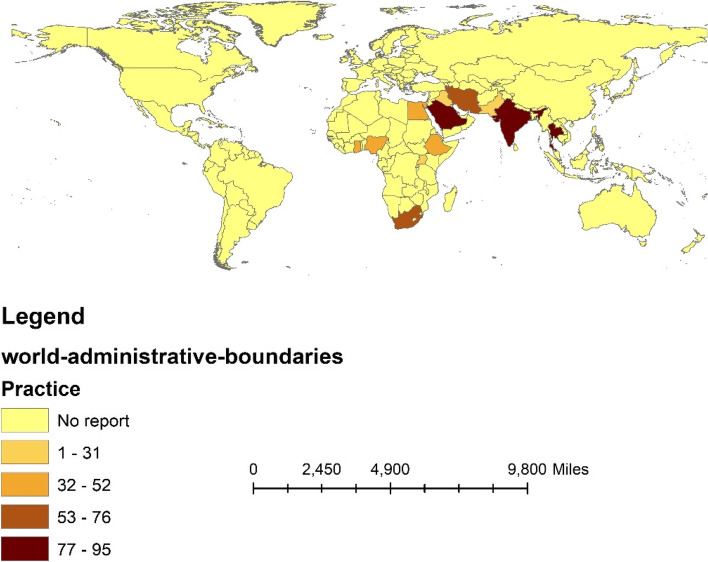
The percentage of appropriate practice towards COVID-19 among pregnant women based on the continent

Subgroup analysis showed that appropriate practice towards COVID-19 in pregnant women in Asia and Africa was estimated to be 60% (95%CI: 41–78) and 50%(95%CI: 39–60) (between-group *p*-value < 0.001), respectively (Table 3 and Fig. 7).

### Publication bias

We employed Egger's regression test and funnel plots to check publication bias. On a condition of confirmation of publication bias, the trim-and-fill method was used to estimate the number of censored studies and finally to correct the overall estimate of the meta-analysis.

**Fig. 8:**
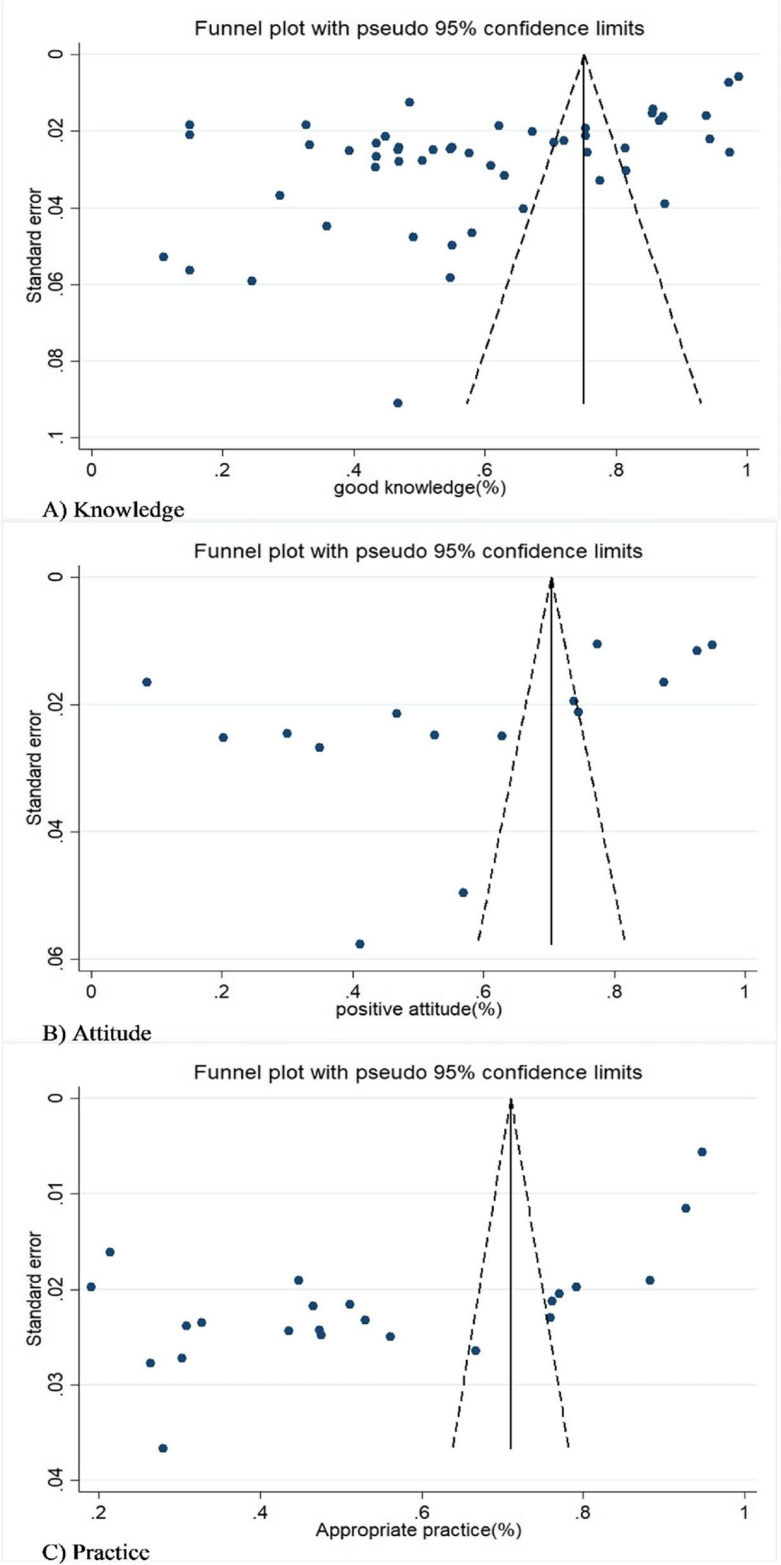
Funnel plot with pseudo 95% confidence limits for detection of publication bias among included studies

The funnel plots and Egger's test showed that there is a significant publication bias for the level of knowledge (bias = -15.8941, 95%CI: -21.322, -10.466, *P* < 0.001), as depicted in Fig. 8, A. Based on the results of trim- and -fill, two studies were censored. Thus, the approximation of the corrected good knowledge level was 58.5%(95%CI: 49.5–67.5%).

Egger's test was significant for good attitude (bias = -21.29213, 95%CI: -39.929, -2.654, P = 0.028). Also, Fig. 8, B displayed that the asymmetric distribution of studies in the funnel plot confirmed the publication bias. Based on the non-parametric method, the trim-and-fill test estimated three censored studies. Consequently, the corrected positive attitude was estimated as 55.6%(95%CI:38.9–72.3%).

For appropriate practice, the funnel plot was asymmetric, and Egger's test was significant (bias = -25.4246, 95%CI: -34.458, -16.39, *P* < 0.001), as shown in Fig. 8, C. Based on Trim-and-fill and non-parametric methods, the expected values of two censored studies were calculated, and the overall appropriate practice corrected by the random effects model in pregnant women was estimated to be 49.4% (95%CI: 33.5–65.3%).

## Discussion

This comprehensive systematic review and meta-analysis study assessed the overall good knowledge, positive attitude, and appropriate practice towards COVID-19 in pregnant women. The study demonstrated that these parameters for COVID-19 infection prevention in pregnant women were low. According to the results, good knowledge was 59%, which was in line with the results of several studies [82–84]. However, this finding is significantly lower than that of research conducted on sub-Saharan Africans [85]. In a systematic review and meta-analysis, Mose et al. estimated that pooled knowledge of COVID-19 infection prevention among pregnant women in Ethiopia was 60.24% [86].

In the present paper, information about knowledge was extracted from 30 studies in Asia (7852 people), 21 studies in Africa (9353 people), and one study in America (114 people). At first glance, it seems that considering that the majority of the study population was conducted from developing countries. The current study's estimate of people's level of knowledge was lower than the real values of the global average, or at least these results cannot be generalized for developed countries.

While taking a closer look at the separate results of different articles, it is revealed that these results have shown the highest knowledge among pregnant women about COVID-19 in African countries (61%), followed by Asian countries (58.8%) and the lowest in the United States of America (35.9%) [29]. Interestingly, the highest knowledge was for an African country (Ghana 85.6%) [68], and the lowest one is for an African country as well (Uganda 32.8%) [75]. Also, Asian countries, such as India (79.5%) (53- 54- 55) and Lebanon (81.5%) [45], showed a high level of knowledge.

In addition, maternal age, educational levels, husband educational levels, underlying disease, and socio-cultural and demographic features were associated with KAP of COVID-19 in pregnant women [22]. Furthermore, a study conducted in China on pregnant women represented that a level of knowledge of COVID-19 prevention related to high education through the media, especially at the beginning of the epidemic, previous experiences of exposure to other coronavirus epidemics, and the local government-imposed strict restrictions on immediate infection control after the outbreak began [31].

The other results of this study demonstrated that the overall positive attitude among pregnant women toward COVID-19 was 57%, which was in line with the results of several studies [82, 83, 87]. A systematic review and meta-analysis estimated that the attitude towards COVID-19 infection prevention among pregnant women in Ethiopia was 62.46%% [86]. In this study, good knowledge of COVID-19 had a better status than a positive attitude. Generally, it is expected that people's knowledge is at a higher level than their attitude. Also, Asia (60%) had a better situation than Africa (52%). However, the highest values of a positive attitude were for Egypt (95%) [57], which has a better socioeconomic status and literacy level than other African countries.

The majority of the studies were online surveys, and literature support, age, and education level affected the behaviors of online surveys [88] since information sources, the Internet, and social networks played an important role in creating knowledge and attitude [89]. Furthermore, the difference in time in terms of the status of the epidemic curve during the study period, as well as the trust in the local government to manage the epidemic, especially the experience of controlling and managing previous epidemics, affected the attitude of the community [22].

Furthermore, our findings showed that the positive practice towards COVID-19 was 53%, which was in line with previous studies [83, 85, 87] and lower than a review conducted around the globe [90]. A systematic review and meta-analysis estimated that the practice among pregnant women in Ethiopia was 52.29% [86]. This study revealed that pregnant women who resided in urban areas were 2.23 times more likely to have good preventive practices for COVID-19 infection compared with those who resided in rural areas. One of the possible reasons may be that urban pregnant women have better access to basic healthcare services and media. They also can read texts related to Covid-19 from newspapers or social media. Moreover, findings showed that pregnant women with a secondary education level perform 3.36 times more preventive behaviors against Covid-19 compared to those with no formal education [86].

On the other hand, it should be mentioned that the present study focused on pregnant women, while the global study conducted worldwide included all people in society. The positive practice towards COVID-19 in pregnant women in the Asian continent was (60%) better compared to the African continent (50%), which seems logical. The level of positive practice of people was lower in knowledge and attitude. Achieving positive practice requires improving knowledge and attitude, yet their improvement does not lead to positive practice in all cases [91]. In a meta-analysis study, Mose et al*.* showed that pregnant women with good knowledge were 2.73 times more likely to have good preventive practices for COVID-19 than those with poor practice [86].

The level of risk perception of society to understand the risk of infection, cultural norms, such as shaking hands and participating in family, social and religious gatherings, continuity of water sources and easy washing of hands, access to the health care facility and living conditions may be effective in carrying out prevention behaviors for COVID-19 in communities [90, 92].

Furthermore, the harm caused by the pandemic may be different in the uninfected pregnant population. In this regard, Zheng et al., in a systematic review and qualitative meta-synthesis study, reported that the COVID‐19 pandemic disrupted the conceiving plan and the routine care of pregnant women. Since the availability and quality of maternal care have played a decisive role in maternal and fetal outcomes, it is suggested that the government or healthcare providers balance the restrictions and access to maternity care during future pandemics [93].

### Strengths and limitations

One of the limitations of the current paper was the lack of studies regarding the KAP components of pregnant women in preventive behaviors against COVID-19, especially in developed countries, which to some extent limited the global estimate of the KAP rate for pregnant women leading to encountering the problem on the comparison of countries and continents. In addition, despite performing meta-regression analysis to find the source of heterogeneity and subgroup analysis to reduce its impact on the estimates, the heterogeneity rate between studies was still high. The reason for this is probably other variables, such as the difference in tools, questionnaires used to measure KAP components, and the difference in the studied societies in terms of basic demographic variables, such as age, literacy level, socioeconomic status, cultural difference, ethnicity, type of health system, and the different policies of the governing systems of the societies to deal with the COVID-19 pandemic in each region and country, which was not investigated in this study. The small sample size of many studies conducted in most countries, which probably cannot be generalized to the population of those countries, is worth considering. In addition, the publication bias among included studies was significant. Despite its correction with statistical methods and the estimation of the number of censored studies, it can still influence the estimates of this study.

However, considering the global estimation of the level of KAP components in pregnant women for COVID-19, we believe that in this study, all the available and accessible information and the appropriate statistical methods have been used for the most appropriate estimation of the KAP components at the global level. Also, by creating scientific evidence, its findings can be used in health policies and prevention programs, especially for possible future epidemics.

## Conclusion

Our results showed that knowledge, attitude, and practice toward COVID-19 infection prevention in pregnant women were low. Considering that several years have passed since the beginning of this pandemic and taking into account the global effects of the disease in terms of health, social, economic, and political, it was expected that the knowledge, attitude, and practice of pregnant women, who are one of the high-risk groups regarding this disease, would be in a better condition. It is proposed that health education programs and empowerment of communities, especially pregnant women, about COVID-19 continue with better planning. For future studies, it is suggested to investigate the KAP of COVID-19 in pregnant women in countries of other continents and geographical regions.

## Supplementary Information

**Additional file 1: S1 Table.** strategy search for the KAP towards COVID-19 among pregnant women.

## Data Availability

The authors acknowledge that data supporting the findings of this study are available in the article [and/or] its supplementary material.

## Abbreviations

KAP: Knowledge, attitudes, and practice
PRISMA: Preferred reporting items for systematic reviews and meta-analyses
WHO: World Health Organization
PROSPERO: International prospective register of systematic reviews
MeSH: Medical Subject Headings
NOS: Newcastle–Ottawa Scale
d.f: Degree of freedom
