# Supplementary material for: Global knowledge, attitude, and practice towards COVID-19 among pregnant women: a systematic review and meta-analysis
Source: BMC Pregnancy Childbirth. 2023 Apr 22;23:278. doi: 10.1186/s12884-023-05560-2 (PMC10122207; doi:10.1186/s12884-023-05560-2)
Supplement: Supplementary file 1 — Additional file 1: S1 Table. strategy search for the KAP towards COVID-19 among pregnant women. [file 12884_2023_5560_MOESM1_ESM.docx]

S1 Table: strategy search for the KAP towards COVID-19 among pregnant women

| **Databases** | **Search terms** |
| --- | --- |
| Google Scholar, Scopus, PubMed/MEDLINE, Science Direct, Web of Science, EMBASE, Springer, and ProQuest | (“knowledge” OR “awareness” OR “attitude” OR “opinions” OR “sentiments” OR “perception” OR “practice”) and( “COVID-19” OR “coronavirus” OR “Corona Virus” OR “SARS CoV-2” OR “Coronavirus Disease 2019” OR”2019 nCoV Infection” OR “pandemic” OR “epidemic”) and (“Pregnant women’). |
| **Number of paper identified** | **Results in each database** |
| 1502 journal papers | Google Scholar=560 papers  PubMed=328 papers  Scopus=215 papers  Embase=106 papers  Web of Science=92 papers  Science Direct=82 papers  ProQuest=68 papers  springer=51 papers |
